# Autism and the criminal justice system: An analysis of 93 cases

**DOI:** 10.1002/aur.2690

**Published:** 2022-03-14

**Authors:** Rachel Slavny‐Cross, Carrie Allison, Sarah Griffiths, Simon Baron‐Cohen

**Affiliations:** ^1^ Autism Research Centre, Department of Psychiatry University of Cambridge Cambridge UK; ^2^ Psychology and Language Sciences University College London London UK

**Keywords:** autism, criminal justice, mitigation, offending, reasonable adjustments

## Abstract

We investigate whether autistic people's vulnerability is taken into account at each stage of the criminal justice system (CJS). Defense lawyers from 12 nations were included in the study although the sample was predominantly from the UK. Lawyers completed an online survey regarding one case in which they had defended an autistic client between January 2015 and January 2020; and on one case in which they had defended a nonautistic client charged with a similar offense, to provide a comparison group. Ninety‐three lawyers (85% in the UK) reported on one autistic case, and 53 also reported on one nonautistic case. 75% of autistic clients were not given reasonable adjustments during the process. Only 43% were offered an appropriate adult during police investigations, even though they had an existing diagnosis of autism. 59% of prosecution barristers and 46% of judges said or did something during the trial that made the lawyers concerned that they did not have an adequate understanding of autism. Lawyers were 7.58 times more likely to be concerned about their autistic client's effective participation in court and were 3.83 times more likely to be concerned that their autistic clients would engage in self‐harm, compared with their nonautistic clients. There is a failure to identify and address autistic peoples' disability within the CJS. There is a need for mandatory autism training for police officers and the judiciary, with a focus on identifying autism and understanding the needs of autistic people so that reasonable adjustments are offered in all cases.

**Lay Summary:**

This study sought to investigate if the needs of autistic people are being overlooked by the police and other professionals within the CJS. Results show that autistic people are not always given the support they need during police questioning or in court. The experience of being involved with the police may also have a more negative impact on autistic peoples' mental health than that of nonautistic people.

## INTRODUCTION

An Equality and Human Rights Commission report published in June 2020 warned that the criminal justice system (CJS) is failing those with learning disabilities, and autistic people. The report concludes that reform is needed to ensure a fair trial for all (Equality and Human Rights Commission, 2020). The literature on supporting people with intellectual disabilities within the CJS is more established. People with intellectual disabilities are over‐represented in police custody and other areas of the CJS (Gulati et al., 2018; Hayes, Shackell, Mottram, Lancaster, 2007). Because of their communication and cognitive difficulties, people with intellectual disabilities are likely to struggle with constructing a clear and consistent narrative of events (Cusack, 2018, 2020), misunderstand their legal rights and the implications of what they say to the police (Erickson, Salekin, Johnson & Doran, 2020; Rogers et al. 2010), and may be more suggestible and more likely to confabulate than those without an intellectual disability (Gudjonsson & Henry, 2003; Gudjonsson & Joyce, 2011). The social and communication difficulties experienced by autistic people can be very different to, and in some cases less obvious than, those with an intellectual disability. Although intellectual disability is common among autistic people (35.2% according to Maenner et al., 2021), the majority of autistic people have an intelligence quotient (IQ) score ≥70 (Maenner et al., 2021), making their social and communication difficulties harder to identify.

There is a lack of available evidence to understand how autistic defendants are being treated at each stage of the CJS. Without such data, it is challenging to identify areas that require reform to ensure justice. Compared with nonautistic individuals, autistic people may have higher rates of interactions with the CJS throughout their lifetime, with a higher number of reports of victimization and assault (Griffiths et al., 2019; Weiss & Fardella, 2018). Autistic people also have an increased likelihood of being arrested and cautioned by police (Griffiths et al., 2019). Most research in this area has focused on autistic peoples' interactions with police (Crane, Maras, Hawken, Mulcahy, & Memon, 2016; Tint, Palucka, Bradley, Weiss, & Lunsky, 2017), with less focus on other stages of the justice process, such as the trial.

Social and communication differences may make navigation of the CJS particularly challenging for autistic people. For example, they may exhibit self‐incriminating behaviors and have more adverse interactions with police (Brewer & Young, 2018). To ensure a defendant is treated fairly, it is reasonable to assume that the system should be adapted to facilitate their effective participation. If the responsibility is placed on the defendant themselves to communicate effectively with different people and provide evidence in court under stressful conditions, then an autistic person may be at a significant disadvantage without adjustments.

In the UK, the Police and Criminal Evidence Act 1984 states that any person suspected of a criminal offense under the age of 18 or a vulnerable adult (i.e., has a disability or mental health condition) must be offered an appropriate adult (AA) during police questioning (Police and Criminal Evidence Act 1984). AAs act to safeguard the interests and rights of vulnerable defendants by ensuring that they are treated in a just manner and are able to participate effectively during an investigation. While AAs are not available in all jurisdictions outside the UK, reasonable adjustments or ‘accommodations’ to usual practices are commonly put in place to address a person's disability across jurisdictions. Under the UK's Equality Act (2010) and the Americans with Disabilities Act (ADA, 1990), all statutory bodies must make reasonable adjustments, or ‘amendments’, to ensure that any individual with a disability is able to fully participate in their criminal proceedings. Adjustments can be made by the police to assist the detainee, such as using visual aids to assist with communication and allowing extra time to process information. Reasonable adjustments may include any steps to encourage and facilitate the participation of the defendant (Ministry of Justice, 2020a; ADA, 1990).

For reasonable adjustments to be made, the detainee must first be correctly identified as a vulnerable adult. Lack of disclosing an autism diagnosis is therefore a major issue: one study found that only 39% of autistic people entering the CJS disclosed their diagnosis to police (Crane et al., 2016). If the detainee does not disclose their diagnosis, they may not be identified as a vulnerable adult and therefore appropriate adjustments will not be made. Adjustments can be crucial for autistic individuals to ensure a fair investigation and trial. The proportion of undiagnosed autistic people entering the CJS is also currently unknown.

There are differences in the structure, terminology, and mechanisms among jurisdictions of different nations. For example, the concept of a ‘vulnerable adult’ is not formally recognized in the American CJS. A person with a disability has rights under the Americans with Disabilities Act of 1990 to ‘reasonable accommodations’, although it is not clear how this is regularly implemented in practical terms. It is important to understand how autistic adults are disadvantaged in the context of different jurisdictions using broadly comparable mechanisms, such as the use of any adjustments to standard procedure to address the specific needs of the defendant.

### Impact on mental health

The impact on an autistic person's mental health and ability to cope with being subjected to a trial is poorly understood. Suicide rates are between 5 and 20 times higher in prisoners compared with the general population (Fazel & Benning, 2009; Fazel, Benning, & Danesh, 2005). Autistic adults have an elevated risk of suicidal ideation and lifetime experience of attempted suicide, which is more than nine times higher than in the general population in England (Cassidy et al., 2014). Autistic people may be a particularly high‐risk population for suicide and self‐harm in custody or during an investigation. To our knowledge no studies have looked at trauma following involvement with the CJS as a defendant, particularly among individuals who are not currently serving a prison sentence. It is reasonable to predict later trauma‐related mental health difficulties following such a highly stressful life event. There are currently no data on suicidality, self‐harm or the associated risk factors in relation to autistic people in custody or subject to a criminal investigation. Such data will assist in the development of suicide prevention strategies, highlighted by the government as a ministerial priority (Harris, 2015; Ministry of Justice, 2020b).

### Aims

The aim of the current research is to collect data from defense lawyers and barristers about autistic people they have represented. By gathering information from legal professionals we address issues such as whether defendants were able to access the support they needed to navigate the CJS (access to fair justice) effectively, the level of autism awareness within the CJS, as indicated by lawyers' satisfaction with the way autistic defendants were treated, and whether the defendant's autism was considered by the judge to be a mitigating factor. Finally, we identify any high‐risk mental health concerns such as self‐harm and suicidality among autistic defendants. The assessment of mental health concerns will be ascertained through the survey of lawyers rather than by assessing autistic defendants directly. We compare autistic and nonautistic cases to examine four key questions across each stage of the justice process (i.e., police investigation, trial, and outcome):

1. Are autistic defendants accessing fair justice?
2. Are reasonable adjustments implemented to enable autistic defendants to participate effectively in their investigation and trial?
3. Are autistic people disadvantaged by the current justice system due to lack of autism awareness?
4. Are autistic defendants at risk of mental ill‐health and suicidality during CJS involvement?

## METHOD

### Study design and participants

Participants were defense solicitors and barristers who had defended an autistic client in the 5 years prior to taking part in the study (since January 2015). Legal professionals were recruited via UK, European and American professional bodies (e.g., the Law Society, the Bar Circuits, European Criminal Bar Association, American Bar Association) and adverts placed on social media (e.g., Twitter, Facebook). A further effort to recruit legal professionals from other countries was made by placing adverts on Reddit, which has a substantial American user base.

A case–control design compared autistic with nonautistic clients of the same lawyer who were charged with a similar category of offense of which a verdict had been reached within the 5 years prior to data collection (after January, 2015). Autistic clients received an autism diagnosis from a medical professional (47 were made by a psychiatrist, 28 by a clinical psychologist, 2 by a neurologist, 4 by a pediatrician, 4 by a GP, 7 were unknown and 1 ‘other’).

Written informed consent was obtained in an online tick box format from all participants. The authors assert that all procedures contributing to this work comply with the ethical standards of the relevant national and institutional committees on human experimentation and with the Helsinki Declaration of 1975, as revised in 2008. All procedures involving human participants were approved by the Psychology Research Ethics Committee, University of Cambridge (PRE.2019.044).

Data were collected using an online survey. Survey questions were developed in collaboration with members of the autistic community with lived experience of the UK CJS to ensure that the content of the survey was relevant. Four autistic adults took part in a focus group and provided written feedback during the design phase and final draft of the survey. Lawyers and barristers were also consulted during the design phase to ensure the content was relevant to legal professionals. Four of the consultants practice in the UK and one practices in both the USA and UK. Survey items were then subdivided into seven sections to improve readability based on feedback from legal professionals: (i) the client (diagnosis, basic demographic information), (ii) the alleged crime (offense category and previous convictions), (iii) the trial (concerns regarding effective participation in court), (iv) the verdict and sentencing, (v) reasonable adjustments, (vi) the legal professional's role in the case, and (vii) concerns regarding the client's mental health during their involvement with the CJS. Data on mental health concerns are not derived by assessing individuals directly but from the accounts provided by lawyers based upon assessments of their client carried out during the justice process. Following consultation with lawyers, it was agreed that the term ‘reasonable adjustments/amendments’ is a familiar legal term to lawyers in the UK and USA, therefore we did not define this term in the survey. Lawyers in other countries may have interpreted the definition of reasonable adjustments differently. All items included a ‘do not know’ response option.

Each section consisted of multiple‐choice questions with ‘do not know’, ‘other (please specify),’ and open text boxes included where appropriate. For items that had a ‘yes’, ‘no’, or ‘do not know’ option, data were scored as 1 for ‘yes’, 0 for ‘no’ and ‘do not know’ was scored as missing data. For Likert scale items (e.g., ‘Overall, how satisfied are you that your client was treated fairly by the CJS?’) data were scored as 1 for ‘extremely dissatisfied’, 2 for ‘somewhat dissatisfied’, 3 for ‘neither satisfied nor dissatisfied’, 4 for ‘somewhat satisfied’ and 5 for ‘extremely satisfied. Survey items can be found in Table S3. To account for the different terminology used between jurisdictions across nations we included definitions alongside items. For example, for the item ‘Was your client offered an AA during police investigations?’, we added ‘This could be an independent person who is there to ensure the needs of a disabled person are met’ to clarify the meaning for non‐UK lawyers.

### Statistical analyses

Statistical analyses were performed using R Version 4.0.2 using the miceadds v3.11‐6 package to run the adjusted standard errors regressions (R Core Team, 2020). Lawyers gave ratings for one autistic defendant and one nonautistic defendant so data were not independent. Therefore, we used regression with clustered standard errors to account for nonindependence within the data. All analyses included group (autism = 1, controls = 0) as an independent variable and lawyer as a cluster variable. Numeric outcome (Likert scale data) were analyzed using linear regression with clustered random errors, while binary outcome measures (‘yes’ or ‘no’) were analyzed using logistic regression with clustered random errors. Confidence intervals (CI; 95%) and odds ratios (OR) were reported for logistic models.

## RESULTS

### Participants

Ninety‐three legal professionals consented to take part in the survey and reported on 93 cases involving autistic individuals. Fifty‐three of the same professionals reported on a nonautistic client they had defended who was charged with a similar offense within the same period (since January 2015). See Table S1 for breakdown of offenses, of which both groups are comparable. Participating legal professionals resided in the UK and Northern Ireland (85%), Pakistan (1%), Afghanistan (1%), Germany (1%), Norway (1%), Hong Kong (1%), Australia (2%), Austria (1%), Croatia (1%), USA (3%), New Zealand (1%) and Argentina (1%). Due to participant drop out, some legal professionals did not complete the entire survey for both autistic and nonautistic clients therefore analyses are run with varying sample sizes. Demographic information about the legal professionals and their clients are summarized in Table 1. Information about clinical diagnoses for all clients is provided in Table S2. There were 4 and 5 female clients in the autism and control groups respectively, therefore we pooled sex across all analyses. Autistic clients were more likely than nonautistic clients to have a diagnosis other than autism, *X* ^*2*^ (1, *N* = 139) = 20·61, *p* > 0·001.

**TABLE 1:** Demographic information for legal professionals and their clients

	Legal professionals *n* = 69	Autism *n* = 93	Control *n* = 53
Age in years			
Mean (SD)	49 (9.92)	26 (11.43)^a^	28 (11.13)^a^
Sex			
Male	46% (32/69)	95% (88/93)	89% (47/53)
Female	54% (37/69)	4% (4/93)	9% (5/53)
Other	0% (0/69)	1% (1/93)	2% (1/53)
Job title			
Barrister	58% (40/69)		
Solicitor	17% (12/69)		
Advocate	16% (11/69)		
Other	9% (6/69)		
Years in practice			
0–5 years	12% (8/69)		
6–10 years	10% (7/69)		
11–20 years	20% (14/69)		
21+ years	58% (40/69)		

^a^ Age at time of alleged offense.

*Note*: Data are n/N (%); due to missing data demographic information is available for 69 legal professionals.

### Access to justice

Only 25% (15/60) of autistic clients were given reasonable adjustments with 38% (23/60) not given any even though lawyers stated that this would have been beneficial. 3% (2/60) stated that their autistic client had not needed adjustments at the police stage while 33% (20/60) did not receive any adjustments because their autism diagnosis was unknown at this time. This means that 75% of autistic clients did not receive any reasonable adjustments at the police station. Of the autistic clients whose case went to trial, 60% (40/67) were given reasonable adjustments in court, and 22% (15/67) were not given any, even though their lawyers stated that this would have been helpful. Only 18% (12/67) were considered not to have needed any adjustments during their trial.

Lawyers were 7·58 (95% CI = 3·75–15·31, *B* = 2·02, *z* = 5·64, *p* < 0·001) times more likely to be concerned about their autistic client's effective participation in court compared with their nonautistic clients. 62% (58/94) received their autism diagnosis before the trial, 29% (27/94) were diagnosed during the criminal proceedings, and 10% (9/94) received their diagnosis after proceedings had ended. These data need to be considered in light of the fact that not all the autistic defendants were diagnosed prior to involvement with the CJS.

### Autism awareness in the CJS

Lawyers were significantly less satisfied with how their autistic clients were treated overall during the justice process, and during each stage of the police investigation (see Table 2). Lawyers reported that just under half of their autistic clients (48%, 39/82) were considered by the police to be vulnerable adults, and a similar percentage (47%, 41/88) were offered an AA during police investigations (see Table 3).

**TABLE 2:** Lawyer satisfaction ratings for treatment of autistic and nonautistic clients

		Autism	Control				
	*N*	*M* (SD)	*M* (SD)	Estimate	SE	*p*	R^2^
Overall	123	2.58 (1.42)	3.40 (1.32)	−0.83	0.26	<0.001	0.08
During arrest	111	2.46 (1.09)	3.38 (0.98)	−0.91	0.19	<0.001	0.16
Whilst held in custody	109	2.54 (1.18)	3.21 (1.05)	−0.67	0.20	<0.001	0.08
During police questioning	111	2.44 (1.29)	3.17 (1.04)	−0.72	0.22	<0.001	0.08

*Note*: Linear regression models with clustered standard errors, group as the independent variable (autistic and control clients) and lawyer is the cluster variable.

**TABLE 3:** Answers to autism awareness questions about autistic clients

	Percentage (n/N)
Was your autistic client considered by the police to be a vulnerable adult?	
Yes	48% (39/82)
No	52% (43/82)
Was your autistic client offered an appropriate adult during police investigations?	
Yes	47% (41/88)
No	35% (31/88)
No, diagnosis was not known	18% (16/88)
Which statement best describes the information given to the jury/tribunal of fact about your client's autism?	
Not informed about client's diagnosis	18% (12/66)
Informed about client's diagnosis but no information beyond this fact was provided	15% (10/66)
Informed about client's diagnosis alongside minimal information about autism (e.g., information not specifically related to your client's needs)	14% (9/66)
Informed about the client's diagnosis alongside sufficient information about autism and how your client's autism may affect their presentation and communication in court	53% (35/66)
The prosecution barrister said or did something that made me concerned that s/he did not have an adequate understanding of autism	
Disagree	21% (15/71)
Neither agree nor disagree	20% (14/71)
Agree	59% (42/71)
The judge/magistrate said or did something that made me concerned that s/he did not have an adequate understanding of autism	
Disagree	39% (28/71)
Neither agree nor disagree	14% (10/71)
Agree	46% (33/71)
How did your client plead?	
Guilty	25% (18/72)
Not guilty	61% (44/72)
Combination (e.g., guilty to lesser offense)	6% (4/72)
Client was not fit to plead	8% (6/72)
What was the outcome of the proceedings?	
Found to have done the act	37% (28/75)
Found guilty of a lesser offense	11% (8/75)
Found not guilty	25% (19/75)
Case was terminated	19% (14/75)
Other	8% (6/75)
What action was taken or what sentence was given?	
None (e.g., found not guilty)	42% (31/74)
Court fine	3% (2/74)
Community sentence	8% (6/74)
Prison sentence	27% (20/74)
Suspended prison sentence	9% (7/74)
Hospital Order	5% (4/74)
Imprisonment for Public Protection (IPP)	3% (2/74)
Other	3% (2/74)
Was your client's autism seen as a mitigating factor by the judge?^a^	
Yes	60% (30/50)
It was considered, but No	22% (11/50)
Client's autism was not considered at all	18% (9/50)
How was your client's autism taken into account?^b^	
Court fine was reduced	7% (2/30)
Length of prison sentence was reduced	33% (10/30)
Prison sentence reduced to community	3% (1/30)
Prison sentence was suspended	23% (7/30)
Discharge	10% (3/30)
Other	23% (7/30)

^a^ Subset of clients who were found guilty of committing a crime.

^b^ Subset of clients whose autism was seen as a mitigating factor.

*Note*: Results are for autistic clients only.

In just over half of the cases that included a trial by jury (53%, 35/66) the jury or tribunal of fact were informed the defendant was autistic alongside information about how this may have affected their presentation and communication in court. Lawyers indicated that 59% (42/71) of prosecution barristers and 46% (33/71) of judges or magistrates said or did something during the trial that made them concerned that they did not have an adequate understanding of autism. In cases where their client was found to have committed a crime, 60% (30/50) of judges saw their client's autism as a mitigating factor, the majority of whom were given a suspended or reduced sentence (see Table 3).

### Mental Ill‐Health and CJS involvement

Lawyers were 3·83 (95% CI = 1·77–8·32) times more likely to be concerned that their autistic clients would engage in self‐harm behaviors compared with their nonautistic clients and were 5·73 (95% CI = 2·46–13·35) times more likely to report that their autistic clients experienced meltdowns as a result of their involvement in the CJS (see Table 4). Group did not significantly predict suicide attempts during clients' involvement with the CJS. Coefficients and standard errors are provided in Table S4.

**TABLE 4:** Percentage of ‘yes’ responses for each mental ill‐health item by group

	Autism	Control		
	% (n/N)	% (n/N)	*p* value	Odds ratio (95% CI)
Since you have known your client, have you ever been concerned that they would self‐harm?	58% (36/63)	27% (13/49)	<0.001	3.83 (1.77, 8.32)
Since you have known your client, have they attempted to end their life?	16% (10/63)	5% (2/41)	0.112	3.75 (0.74, 19.10)
Has your client experienced meltdowns as a result of their involvement in the criminal justice system?	68% (42/62)	27% (11/41)	<0.001	5.73 (2.46, 13.35)

*Note*: Results are from logistic regression (with clustered standard errors), fixed effect of group, lawyer is the cluster variable.

## DISCUSSION

This study provides novel data on whether autistic defendants are treated with full consideration regarding their disability during involvement with the CJS. We also sought to document the impact the justice process has on autistic defendants' mental health. Our data has highlighted several injustices at each stage of the CJS; from police arrest through to a trial. This study uniquely explores these key issues by collecting data from those in the defendant's legal team.

The first aim of this study was to identify barriers to autistic defendants accessing a fair trial. We predicted a lack of autism awareness within the CJS, both at a systemic and individual level. It is a requirement of the CJS in England and Wales that all defendants have a right to a fair trial. This means that those with a disability and/or mental health condition should be identified from the start of the justice process and adjustments made to ensure that the defendant understands and can effectively participate in an investigation and trial. Atypical behavior and the communication style of autistic people may be misinterpreted by the police, legal team, jurors and the judge (Freckelton, 2011). Therefore, autism awareness and acceptance of difference is particularly pertinent to a fair CJS.

This study identifies a systemic barrier in the identification of autistic people as vulnerable adults if they enter the CJS. In this study, 35% (31/88) of autistic defendants were not given an AA during police investigations and a further 18% (16/88) did not have an AA present because their diagnosis was not known to the police. The use of AAs to assist vulnerable adults at the police station is only available in the UK, so these results are not generalisable to other jurisdictions. However, the concept of reasonable adjustments is a requirement under the ADA (1990) in the USA and the Equality Act (2010) in the UK. The identification of vulnerable defendants is a vital first step in ensuring that their right to a fair investigation is upheld. Our data suggest that a proportion of autistic defendants are not identified as vulnerable adults and that this is even evident in cases where an existing diagnosis is present. Shockingly, 38% (23/60) of the autistic defendants in this study were not given any reasonable adjustments at the police station even though their lawyers stated that this would have been beneficial, and 33% (20/60) did not receive any adjustments during police questioning because their autism diagnosis was not known at the time. The current literature on intellectual disabilities and policing demonstrates a more general, global problem with accurately identifying vulnerable people within the CJS (Gulati, Kelly, Cusack, Kilcommins & Dunne, 2020). However, the focus of this paper was to identify the needs of autistic people rather than to establish needs that are unique to autistic people compared with those who have other social and communication difficulties. There are specific needs of autistic individuals that may not necessarily be the same as those with other disabilities. For example, autistic people may have specific sensory difficulties that may need special consideration during police contact (i.e., reduction of background noise and not being touched unless absolutely necessary). The needs of autistic individuals who do not have a learning disability may more likely go unidentified and unmet if they have fluent language and average‐range intellectual ability. It is also these individuals who tend not to be identified as autistic in childhood and enter adulthood without a diagnosis and support in place (Baron‐Cohen et al., 2009; Lai & Baron‐Cohen, 2015).

Lawyers were also significantly more likely to be concerned about their autistic client's effective participation in court compared with their nonautistic clients. Additionally, 60% (40/67) of autistic clients who had a trial were given reasonable adjustments in court compared with only 25% (15/60) at the police station suggesting that more autistic defendants were given reasonable adjustments in court than when they were in police custody. These findings could be a reflection of the lawyers and barristers in our sample being more experienced at identifying autistic defendants' support needs once they are instructed, which is typically following a police investigation. Autistic clients may need reasonable adjustments at every stage of the CJS to ensure that they are able to participate effectively in the justice process. Our research suggests a failure to identify all autistic defendants as vulnerable adults as they enter the CJS, which undoubtedly leads to poor access to justice.

Identifying a defendant as autistic and as a vulnerable adult is the first step in ensuring effective participation throughout the CJS. There are three possible barriers to identification: First, many autistic people do not disclose their diagnosis at the point of police contact (Crane et al., 2016). Second, undiagnosed autistic people may only be identified by legal professionals who have enough experience to identify social and communication difficulties promptly and seek an autism assessment for the defendant. In the current sample, 29% (27/93) received their autism diagnosis during criminal proceedings and 10% (9/93) after proceedings. A diagnosis before, or early in the justice process would undoubtedly lead to better outcomes in terms of access to justice. Finally, even autistic defendants who disclose their diagnosis are failing to receive reasonable adjustments. Only 25% (15/60) of autistic defendants were given reasonable adjustments despite their known vulnerability. This is compared with 38% (23/60) who were not given adjustments even though their lawyers stated that this would have been beneficial. These data suggest that there is an urgent need for police officers and legal professionals to receive mandatory autism awareness training to alert them to identify autistic traits so that they can access the support they may need as vulnerable adults. NICE recommended that a measure such as the Autism Spectrum Quotient‐10 (Allison, Auyeung, & Baron‐Cohen, 2012) may support the identification of autistic adults with additional needs as they enter police custody.

Lawyers were significantly more satisfied with how their nonautistic clients were treated by the CJS than their autistic clients were at each stage of the process. 59% (42/71) of prosecution barristers and 46% (33/71) of judges said or did something during the trial that made the defense lawyers concerned that they did not have an adequate understanding of autism. In only about half of the cases that had a trial by jury, the jury were informed that the defendant was autistic alongside information about how this may have affected the defendant's presentation and communication in court. Not giving jurors adequate information about a defendant's autism may lead to jurors misinterpreting the autistic behavior of a defendant in court. Education about autism for judges and jurors would likely avoid discriminatory biases arising from ‘odd’ or atypical behavior exhibited by the defendant. Previous research has shown that providing mock jurors with diagnostic information about an autistic witness affected credibility ratings (Crane et al., 2020). More research is needed to understand how jurors appraise autistic defendants' in terms of their credibility when giving evidence in court and what level of information may mitigate biased judgments relating to their presentation.

### Implications for mental ill‐health

The prevalence of co‐occurring mental health problems such a stress and heightened anxiety among autistic adults is high (Nah, Brewer, Young, & Flower, 2018; Park et al., 2019), with many autistic people experiencing meltdown and shutdown as a result (Robertson et al., 2018). Previous research in this area is sparse but one study identified that many autistic people's interactions with the police in Canada occurred in the context of a mental health crisis, with some defendants describing having experienced meltdowns, extreme anxiety and panic preceding their police involvement (Salerno & Schuller, 2019). Autistic clients in our sample were more likely than controls to have a co‐occurring mental health condition (such as depression or anxiety) which may have also shaped their interactions with the CJS and their ability to cope with the stress of being subject to criminal proceedings.

Self‐harm is high among people in custody (Favril, Indig, Gear, & Wilhelm, 2020; Fazel, Hayes, Bartellas, Clerici, & Trestman, 2016), and autistic people are a high‐risk group for self‐harm, with 66% reporting suicidal ideation (Cassidy et al., 2014). Little research has looked at self‐harm and suicidality among autistic people in prison but one study has shown that prisoners with neurodevelopmental difficulties are more likely to have thoughts about self‐harm and suicide (McCarthy et al., 2019). To our knowledge no data currently exists on how autistic defendants cope with the stress of an investigation and court proceedings. This is a particularly important area of research as unmet support needs and nonsuicidal self‐injury predicts suicidality in autistic people (Cassidy, Bradley, Shaw, & Baron‐Cohen, 2018).

Our findings show that lawyers were nearly four times more likely to be concerned that their autistic clients would engage in self‐harm behaviors compared with their nonautistic clients and were nearly six times more likely to report that their autistic clients experienced meltdowns as a result of their involvement in the CJS. However, autism diagnosis did not significantly predict whether the client had attempted to end their life in the time that the lawyer had known them. Given the small sample size, this result should be interpreted with caution. Overall, these data are concerning and more attention is urgently needed to assess poor mental health and the risk of self‐harm among autistic people as they navigate the CJS. There has been mounting pressure on all UK government organizations (e.g., the Ministry of Justice, Her Majesty's Prison & Probation Service) to address shocking reports on the incidence of suicide and self‐harm in prisons. A Council of Europe (2020) report showed that a person in prison takes their life every 5 days, and in the 12 months preceding the report in 2019 self‐harm incidents reached a record high up by 14% from the previous 12 months (Council of Europe, 2020). Reports published by the U.S. Department of justice reveal similar concerns regarding suicide in prisons (Department of Justice, 2020). Hitherto, there is no body of research that examines the self‐harm and suicide risk among autistic defendants. More research is needed to further quantify this risk and identify ways in which vulnerable defendants can be supported throughout the justice process.

### Policy implications

A key policy implication of this study is the importance of autism awareness training across the CJS. Autism awareness training for CJS professionals would help staff identify more subtle social and communication difficulties and equip professionals with practical advice on how to adjust practices to support autistic people. Some states in the USA, such as New Jersey (Kelly & Hassett‐Walker, 2016) and Pennsylvania (Act 25, 2015), have mandatory autism‐awareness training for law enforcement officers, but the majority of states do not cover autism in their law enforcement training programmes. Criminal justice training is also available in the UK, run and accredited by the National Autistic Society. These include brief online modules and more in depth workshops (for more details, see https://www.autism.org.uk/advice-and-guidance/topics/criminal-justice/criminal-justice/professionals). Some police services have opted in to the National Autistic Society's autism‐awareness training, however this is not mandatory and many CJS professionals in the UK receive no training in autism. Our data strongly suggests that all police services should have mandatory autism training, with a focus on identifying autistic traits. Screening tools may also help police to identify when someone might be vulnerable in the absence of a diagnosis. However, we do acknowledge the limited resources available to police services and the CJS more broadly, which may make implementing screening measures difficult.

There is also a clear need for autism awareness training within the judiciary. Judiciary training should include how to ensure the entire courtroom is autism aware to facilitate effective participation for the defendant. This may include adjustments to court procedures and instructions to jurors so that they have full understanding of a defendant's disability and how it may affect their communication in court. The impact for an autistic defendant of receiving legal counsel from an autism aware legal team may be far reaching. First, it may better enable effective communication between client and lawyer. Second, the defense team may be better placed to put forward a case for mitigating circumstances and to arrange reasonable adjustments on behalf of their client.

Alternatives to prosecution should be considered as a first point of action where appropriate. Diverting people away from prosecution using court ordered community sentences has been shown to be more effective in reducing reoffending than short prison sentences (Ministry of Justice, 2019). Community‐based offender programmes must be adapted to suit the needs of autistic people. Where custodial sentences are necessary, prisons and probation services should take extra measures to adapt their services for autistic individuals. The UK's National Autistic Society has worked with Her Majesty's Young Offender's Institution (HMYOI) Feltham to develop Autism Accreditation standards for prisons (Hughes, 2019). These standards involve the inclusion of an autism ‘champion’ among staff, autism training programme for staff, a plan for each autistic person covering their unique triggers, strengths, weakness and support needs, and a commitment to making reasonable adjustments to ensure that the prison environment and activities are accessible to autistic people (e.g., allowing the use of ear defenders, helping autistic people to become familiar with rooms and activities prior to starting workshops). This work should be used as a blueprint for gold standard practice and more pressure should be placed on prisons to adopt these measures where possible.

While different nations vary in terms of jurisdiction and legal requirements to address vulnerability and disability within the justice system, it is informative to gather data on the broader concepts that apply to varying jurisdictions such as overall satisfaction with treatment by the CJS, whether a vulnerability was recognized, what adjustments were made, and if the autistic person was affected by self‐harm and suicidality during their CJS involvement. Future research should highlight disadvantages specific to different legal jurisdictions and determine whether certain legal practices are working well or less so.

### Limitations and conclusions

There are a number of limitations with this study. The data rely on the accurate reporting of legal professionals. To improve accuracy, we asked professionals to report cases that reached a verdict within the last 5 years in order to include recent cases and current jurisdictions. Lawyers were also asked to rely on their extensive case notes and files on the cases they reported on. It should also be noted that the legal professionals who took part in the study may have a particular interest in autism and so may be a subset of lawyers who are better at identifying autistic defendants and their support needs. Alternatively, lawyers may have been more sympathetic when answering questions about a known minority group. This study presents preliminary data on the disadvantages experienced by autistic people in comparison to nonautistic people, which may be useful for policy makers and legal professionals who work with autistic people. However, these data do not elucidate whether and how autistic people are being disadvantaged compared with other minority groups. The sampling strategy was largely UK‐focused which reflected the proportion of UK‐based lawyers taking part (85%). While steps were taken to address this limitation, such as keeping items broad and relevant to multiple jurisdictions and recruiting lawyers from social media with an international usership, it is nevertheless important to note that the implications of these findings may not be relevant to other nations with differing CJS structures.

Autistic clients included in this study were more likely to have a co‐occurring mental health condition than nonautistic clients. It is difficult to be sure which problems these clients faced were due to their autism and how much stems from their co‐occurring mental health conditions. However, the current study presents real‐world cases where a high rate of co‐morbidity is expected, particularly given that living with autism increases ones' risk of depression and anxiety (Nah, Brewer, Young, & Flower, 2018; Park et al., 2019). It is not clear that splitting these diagnoses will tell us more about the impact of these conditions in cases where they co‐occur.

These data provide an insight into the injustices autistic people face following arrest and identifies a number of situations at each stage of the justice process in which there is a failure to identify, acknowledge and address autistic peoples' disability. Policy changes are needed to address issues such as the systematic identification of autism among police detainees and widespread autism awareness training for police officers, the Judiciary, and other legal professionals. Data are needed from autistic defendants themselves to fully understand their experiences at each stage of the CJS and its impact on their mental health. There is currently no evidence that autistic people are more likely to offend than nonautistic people. Data are needed on whether autistic people are more likely to be noticed by law enforcement and are more vulnerable to offending. Whilst the data presented here are preliminary, it is hoped that this study will be a catalyst for future research in this area. An important area for future study includes understanding how autistic people who are victims of crime navigate the CJS and whether their support needs are met.

## Supporting information


**Table S1** Offense categories for autistic and control clients
**Table S2**. Diagnoses given by a clinician for autistic and control clients
**Table S3**. Coefficients and standard errors for regressions shown in Table 6
**Table S4**. Lawyers survey questions
