# Supplementary material for: Autism and the criminal justice system: An analysis of 93 cases
Source: Autism Res. 2022 Mar 14;15(5):904–14. doi: 10.1002/aur.2690 (PMC9314022; doi:10.1002/aur.2690)
Supplement: Supplementary file 1 — Table S1 Offense categories for autistic and control clients Table S2. Diagnoses given by a clinician for autistic and control clients Table S3. Coefficients and standard errors for regressions shown in Table 6 Table S4. Lawyers survey questions [file AUR-15-904-s001.docx]

**Supplementary Table 1.** **Offence categories for autistic and control clients**

|  | **Autism** | **Control** |
| --- | --- | --- |
| **Fraud, cybercrime, hacking** | 2% (2/93) | 2% (1/53) |
| **Harassment** | 4% (4/93) | 0% (0/53) |
| **Sexual offences** | 34% (32/93) | 38% (20/53) |
| **Less serious violent offences (e.g. common assault)** | 15% (14/93) | 13% (7/53) |
| **Serious violent offences (e.g. wounding with intent, murder)** | 23% (21/93) | 23% (12/53) |
| **Theft or robbery** | 1% (1/93) | 2% (1/53) |
| **Vandalism including arson** | 3% (3/93) | 0% (0/53) |
| **Drug offences** | 2% (2/93) | 9% (5/53) |
| **Possession of weapon** | 4% (4/93) | 2% (1/53) |
| **Terrorism offences** | 4% (4/93) | 2% (1/53) |
| **Other offences** | 6% (6/93) | 9% (5/53) |

Note. Data are n/N (%), for which n is the number of clients charged with the offence and N is the number of clients in the group.

**Supplementary Table 2. Diagnoses given by a clinician for autistic and control clients**

|  | **Autism**  **n = 88** | **Control**  **n = 51** |
| --- | --- | --- |
| **No diagnoses (†)** | 27% (24/88) | 67% (34/51) |
| **Intellectual disability** | 23% (20/88) | 9% (5/51) |
| *Mild* | 5% (4/88) | 0% (0/51) |
| *Moderate* | 15% (13/88) | 8% (4/51) |
| *Severe* | 3% (3/88) | 2% (1/51) |
| **Mental health disorder** | 42% (37/88) | 16% (8/51) |
| *Depression* | 31% (27/88) | 14% (7/51) |
| *Anxiety disorder* | 34% (30/88) | 6% (3/51) |
| *Conduct disorder* | 6% (5/88) | 0% (0/51) |
| *Obsessive compulsive disorder* | 11% (10/88) | 2% (1/51) |
| *Schizophrenia/Psychosis* | 2% (2/88) | 2% (1/51) |
| *Post-traumatic stress disorder* | 1% (1/88) | 0% (0/51) |
| *Bipolar disorder* | 1% (1/88) | 0% (0/51) |
| *Personality disorder* | 1% (1/88) | 0% (0/51) |
| **Neurodevelopmental disability** | 33% (29/88) | 6% (3/51) |
| *ADHD* | 28% (25/88) | 0% (0/51) |
| *Dyslexia* | 5% (4/88) | 0% (0/51) |
| *Dyspraxia* | 2% (2/88) | 0% (0/51) |
| *Foetal valproate syndrome* | 1% (1/88) | 0% (0/51) |
| **Physical disability** | 5% (4/88) | 6% (3/51) |

†Other than an autism spectrum condition for clients in the autism group

Note: Data are n/N (%); Due to missing data diagnoses information is available for 88 autistic and 51 control clients.

**Supplementary Table 3.** **Coefficients and standard errors for regressions shown in Table 6**

|  | **Estimate** | **Std. Error** | ***p* value** |
| --- | --- | --- | --- |
| **Since you have known your client, have you ever been concerned that they would self-harm?** | 1.34 | 0.40 | *<* 0.001 |
| **Since you have known your client, have they attempted to end their life?** | 1.32 | 0.83 | 0.112 |
| **Has your client experienced meltdowns as a result of their involvement in the criminal justice system?** | 1.75 | 0.43 | *<* 0.001 |

Note. Logistic regressions with clustered standard errors, with group as the dependent variable and lawyer as the cluster variable*.*

**Supplementary Table 4.** **Lawyers** **survey questions**

|  | **Lawyers survey questions** | **Answer format** |
| --- | --- | --- |
| **1** | Does this client have an autism diagnosis (diagnosed by a medical professional) | Binary choice |
| **2** | When did they receive their diagnosis in relation to the criminal proceedings? | Multiple choice |
| **3** | At what point did your client disclose their autism diagnosis? | Multiple choice |
| **4**† | Did your client have any [other] disability? | Multiple choice |
| **5**† | If ‘intellectual disability’ ticked: | Multiple choice |
|  | What type of intellectual disability did your client have? |  |
| **6**† | If ‘mental health condition’ ticked:  What mental health condition/s did your client have? | Multiple choice |
| **7**† | If ‘neurodevelopmental condition/s’ ticked: | Multiple choice |
|  | What neurodevelopmental condition/s did your client have (other than autism)? |  |
| **8**† | Was your client considered by the police to be a vulnerable adult? | Binary choice |
| **9**† | Was your client offered an appropriate adult during police investigations? | Multiple choice |
| **10**† | How old was your client at the time of the alleged crime? | Multiple choice |
| **11**† | What was your client's sex?† | Multiple choice |
| **12**† | In which category did your client's alleged offence fall? | Multiple choice |
| **13**† | Did you have any concerns regarding your client's effective participation in court? | Binary choice |
| **14** | Please indicate how much you agree or disagree with the following statements about your client's trial: | Likert scale |
|  | i) The prosecution barrister said or did something that made me concerned that s/he did not have an adequate understanding of autism |  |
|  | ii) The judge/magistrate said or did something that made me concerned that s/he did not have an adequate understanding of autism |  |
| **15** | Which statement best describes the information given to the jury/tribunal of fact about your client's autism? | Multiple choice |
| **16**† | How did your client plead? | Multiple choice |
| **17**† | What was the outcome of the proceedings? | Multiple choice |
| **18**† | What action was taken or what sentence was given? | Multiple choice |
| **19** | Was your client's autism seen as a mitigating factor by the judge? | Multiple choice |
| **20** | How was your client's autism taken into account? | Multiple choice |
| **21**† | How satisfied are you regarding the way your client was treated by police in the following circumstances?: | Likert scale |
|  | During the arrest |  |
|  | Whilst held in police custody |  |
|  | During police questioning |  |
| **22** | Were any reasonable adjustments made at the police station in light of your client's autism diagnosis? | Multiple choice |
| **23** | Were any reasonable adjustments made in court in light of your client's autism diagnosis? | Multiple choice |
| **24**† | Overall, how satisfied are you that your autistic client was treated fairly by the Criminal Justice System? | Likert scale |
| **25**† | Since you have known your client, have you ever been concerned that they would self-harm? | Binary choice |
| **26**† | Since you have known your client, have they attempted to end their life? (as far as you know) | Binary choice |
| **27**† | Has your client experienced meltdowns as a result of their involvement in the criminal justice system? | Binary choice |
| **28** | What is your age? | Multiple choice |
| **29** | What is your gender? | Multiple choice |
| **30** | What is your job title? | Multiple choice |
| **31** | For how many years have you worked as a solicitor, barrister or legal representative? | Multiple choice |

†Questions were repeated for the non-autistic client

Note: The survey presented to lawyers included questions for a separate study and so only those use in this analysis are included in the table. We expected the majority of our participants to reside in the UK and USA so we adapted the survey using American legal terminology (e.g. ‘attorney’ instead of ‘lawyer’) for participants who stated that their country of residence was the USA. The table displays the UK wording of the survey questions.
